# Evaluating the Cyclical Hybrid Imputation Technique (CHIT) under MCAR, MAR, and MNAR missing data mechanisms: Evidence from health datasets

**DOI:** 10.1371/journal.pone.0359577

**Published:** 2026-09-29

**Authors:** Kurban Kotan, Serdar Kırışoğlu

**Affiliations:** 1 Department of Artificial Intelligence and Machine Learning, Faculty of Computer and Information Sciences, Manisa Celal Bayar University, Manisa, Turkey; 2 Department of Computer Engineering, Engineering Faculty, Duzce University, Düzce, Turkey; Takushoku University, JAPAN

## Abstract

Choosing an appropriate imputation strategy for clinical datasets requires understanding which missing data mechanism is operative, yet most imputation benchmarks conflate performance across mechanisms. The present work addresses this gap by conducting the first mechanism-stratified comparison of three imputation approaches—CHIT, Multiple Imputation by Chained Equations via BayesianRidge (MICE), and Random-Forest-based Iterative Imputation—across Missing Completely at Random (MCAR), Missing at Random (MAR), and Missing Not at Random (MNAR) conditions. Three health datasets serve as experimental platforms: the Chronic Kidney Disease (CKD) dataset, the Heart Disease Dataset (HDD), and the Mice Protein Expression Dataset (MPED). Domain-knowledge-driven missingness patterns are constructed for each mechanism (Fig 2) at approximately 20–25% overall rates. Eight classifiers—KNN, Logistic Regression, SVC, Decision Tree, Random Forest, Gaussian Naïve Bayes, MLP, and a deep neural network—are trained on imputed data following GridSearchCV optimisation. Across all conditions, CHIT achieves near-perfect or perfect downstream classification, with SVC reaching up to 100% accuracy on the CKD dataset under MNAR, and 98.75% under MCAR and MAR. Competing methods degrade by up to 19.38 percentage points under MNAR, while CHIT’s accuracy remains stable. Two structural properties account for this resilience: an iterative enrichment of the regression training set as records are completed, and a within-record prioritisation of the most data-scarce features for model-based filling. Taken together, these results provide mechanism-specific guidance for imputation selection in health informatics pipelines. Statistical significance of classifier accuracy differences between CHIT and MICE was assessed using McNemar’s test (two-tailed, continuity-corrected chi-square) applied to the exact binary prediction vectors from each experiment [n_test = 80]. Ninety-five percent confidence intervals for all reported accuracy values were computed using the Wilson score method [z = 1.96]. The 42-configuration mean accuracy and standard deviation for CHIT are reported in Supplementary Table S1. Full McNemar results with confidence intervals are in Supplementary Table S2.

## 1 Introduction

Incomplete records are endemic to clinical data. Large retrospective analyses consistently report missing-value rates between 15% and 50% in electronic health records [1,2], with the extent and pattern of missingness varying by institution, clinical domain, and data-entry practice. The consequences for downstream modelling are well-documented: list-wise deletion introduces selection bias [3,4]; mean and median substitution attenuate variance and distort inter-feature correlations [4]; and even principled multiple-imputation frameworks can produce biased estimates when their model assumptions are violated [5,6]. In health informatics, where predictive models directly inform diagnosis and treatment decisions, these imputation errors propagate into clinically consequential classification mistakes.

A foundational insight from Rubin [7] is that no single imputation strategy is universally optimal: the appropriate method depends on the mechanism governing why values are absent. Rubin’s taxonomy distinguishes three mechanisms. Under Missing Completely at Random (MCAR), missingness is independent of all observed and unobserved data—a strong assumption rarely satisfied in clinical practice but useful as a theoretical baseline. Under Missing at Random (MAR), absence depends on observed variables but not on the unobserved value itself; this is the standard assumption underlying MICE and most multiple-imputation frameworks [6]. Under Missing Not at Random (MNAR), missingness depends on the missing value itself—the most challenging case and arguably the most prevalent in clinical data [3,7], where extreme measurements, non-attendant patients, and systematic reporting omissions generate structured, non-ignorable gaps [5,6].

CHIT, introduced in [8], addresses missing-value imputation through a two-stage iterative design: incomplete records are first partially filled using distributional statistics of already-complete records (column stage), and the resulting temporary estimates are then refined using supervised regression models trained on those same complete records (row stage). On three health benchmarks with naturally occurring missingness, this design surpassed both MICE and RF-Iterative Imputation, yielding perfect classification accuracy for multiple classifier–imputation pairings [8]. A critical limitation of that study, however, is that the missingness mechanism was neither controlled nor characterised: it remains unknown whether CHIT’s superiority is universal across MCAR, MAR, and MNAR or whether it is confined to the specific missingness structure present in the original datasets.

The present paper closes this gap. We design three controlled missingness scenarios—MCAR, MAR, and MNAR—grounded in clinical domain knowledge for each dataset, and evaluate CHIT against MICE and RF-Iterative Imputation under each condition. The MNAR simulation for the CKD dataset removes haemoglobin values below the 12 g/dL anaemia threshold, mirroring real-world informative censoring in nephrology [9,10]. The MAR simulation uses hypertension status to govern blood pressure missingness, reflecting the documented tendency for hypertensive patients to have more complete blood pressure records [2]. The MCAR simulation applies a uniform random mask as a mechanism-free reference. All eight classifiers are hyperparameter-tuned with GridSearchCV, replicating the optimisation protocol of our original CHIT study.

The remainder of this paper is structured as follows. Section 2 surveys related work on missing-data mechanisms, mechanism-aware imputation, and CKD/HDD classification. Section 3 describes datasets, preprocessing, and the three missingness simulation protocols. Section 4 recapitulates CHIT with mechanism-specific analysis. Section 5 presents results. Section 6 discusses findings. Section 7 concludes.

## 2 Related work

### 2.1 Missing data mechanisms and clinical prevalence

Rubin’s [11] three-way taxonomy established the theoretical framework for missing-data analysis, and Little and Rubin [6] provided its full statistical treatment. The key practical distinction is between ‘ignorable’ mechanisms (MCAR and MAR), under which valid likelihood-based inference can proceed without modelling the missingness process, and ‘non-ignorable’ MNAR, which requires explicit joint modelling of data and missingness [5]. Seaman and White [11] demonstrated through simulation that even modest MNAR departures from MAR bias multiple-imputation point estimates and standard errors, with the bias growing with the fraction of missing data.

In clinical settings, MNAR is pervasive. Babitt and Lin [9] documented the mechanistic basis of anaemia in CKD, showing that severely anaemic patients have systematically incomplete haematological records. Tangri et al. [10] found informative censoring of eGFR in CKD progression studies. Marston et al. [2] showed that systolic blood pressure is less often recorded in non-hypertensive patients, providing a clinically grounded MAR pattern. Donders et al. [1] estimated that fewer than 5% of clinical datasets satisfy strict MCAR, making MCAR primarily a methodological benchmark rather than an operational assumption. Sterne et al. [4] further emphasised that incorrect assumption of MCAR when data are actually MAR or MNAR leads to biased and inefficient inferences in clinical research.

### 2.2 Traditional imputation methods

Simple substitution methods—mean, median, mode, forward/backward fill, and interpolation—are computationally trivial and ubiquitous in preprocessing pipelines but are known to produce biased results when the MCAR assumption is violated [4,12]. Van Buuren and Groothuis-Oudshoorn [13] developed MICE, which constructs a sequence of conditional regression models and has become the de facto standard for multiple imputation. Its scikit-learn implementation as IterativeImputer with a BayesianRidge estimator [14] provides automatic regularisation through evidence maximisation, making it robust to collinear clinical features. However, MICE assumes MAR; its performance degrades under MNAR unless the imputation model is augmented with auxiliary variables that explain the missingness [5,6,11,15].

KNN-based imputation [16], introduced for microarray data, fills missing values by averaging over the k nearest complete observations. Acuna and Rodriguez [17] extended its application to clinical datasets and showed its superiority over mean substitution under MAR. Waljee et al. [18] validated KNN imputation on electronic health records and found it superior to mean and regression imputation for MAR data but noted instability when extreme values were systematically absent—a pattern characteristic of MNAR. Interpolation methods, while appropriate for ordered or time-series data, provide no theoretical advantage for cross-sectional clinical records [12].

### 2.3 Machine-learning-based iterative imputation

Stekhoven and Bühlmann’s MissForest [19] demonstrated that Random Forest regressors outperform parametric iterative methods on mixed-type datasets, a finding replicated by Waljee et al. [18] and confirmed in broader benchmark evaluations by Jager et al. [20]. Gondara and Wang [21] proposed MIDA (Multiple Imputation via Denoising Autoencoders), extending deep generative models to the imputation task and showing gains under MAR for high-dimensional data. Nazabal et al. [22] further extended variational autoencoders to heterogeneous missing data, achieving competitive MNAR performance. These deep generative approaches require substantially larger datasets and more computational resources than tree-based methods, limiting their applicability to small clinical datasets such as the CKD dataset.

HyperImpute [23], proposed by Jarrett and colleagues, dynamically assigns a separate prediction model to each feature at each imputation round, yielding strong empirical performance across diverse benchmarks. HIMP [24] takes a pattern-partitioning perspective, maintaining distinct imputation sub-models for each observed missingness configuration. While [8] draws on similar ensemble-regression intuitions, it departs from both frameworks by imposing an explicit ordering on which incomplete records to fill first (those with the fewest gaps) and, within each record, which features to target first (those with the globally highest missingness rate across the dataset). Neither HyperImpute nor HIMP incorporate these dual ordering constraints.

### 2.4 Mechanism-specific benchmarks

Surprisingly few studies evaluate imputation methods across all three Rubin mechanisms simultaneously. Schafer and Graham [25] provided theoretical comparisons but no empirical benchmark. Emmanuel et al. [26] surveyed the machine-learning imputation landscape [27] and identified mechanism-specific evaluation as a key gap. Jager et al. [20] compared seven imputation methods on 13 binary classification datasets under MCAR and MAR but did not include MNAR. Shadbahr et al. [28] similarly omitted MNAR from their comparative benchmark. Twala et al. [29] studied classifier robustness to imputation errors under different mechanisms but focused on decision trees only. The present study is, to our knowledge, the first to evaluate a cyclical hybrid imputation algorithm across all three mechanisms on multiple health datasets with clinically motivated simulation protocols.

### 2.5 CKD and heart disease classification

The UCI CKD dataset [30] is a standard benchmark for both imputation and classification research. Qin et al. [31] applied KNN imputation followed by six classifiers, with their integrated Random Forest–Logistic Regression model achieving 99.83% accuracy. Khalid et al. [32] proposed a hybrid ensemble (Gaussian Naïve Bayes, Gradient Boosting, Decision Tree with Random Forest meta-classifier) and achieved 100% accuracy. Dritsas and Trigka [33] applied Gradient Boosting on the same dataset and reported 99.80% accuracy. For the Heart Disease Dataset, Chandrasekhar and Peddakrishna [34] achieved 93.44% accuracy using Logistic Regression and soft voting ensemble. None of these studies controlled for the missingness mechanism or reported performance stratified by MCAR, MAR, and MNAR, which is precisely the contribution of the present work.

Erol et al. [35] analysed the joint effect of preprocessing technique and classifier on COVID-19 diagnosis, finding that preprocessing (including imputation) accounts for a statistically significant share of downstream performance variance. García-Laencina et al. [36] showed that SVMs are particularly sensitive to imputation quality because support vector selection depends on feature values near the decision boundary. Jerez et al. [37] found that imputation method advantages grow with the proportion of missing data. Graham [38] similarly documented that imputation method selection measurably affects downstream classifier performance, particularly for complex models. These findings provide theoretical grounding for the classifier-specific patterns we report in Section 5.

## 3 Materials and methods

### 3.1 Datasets

**Chronic Kidney Disease (CKD) Dataset [**31**]:** 400 patient records with 24 clinical features (14 continuous, 10 categorical) and a binary class label (CKD / not-CKD). The dataset contains substantial natural missingness across 14 features, with haemoglobin missing in 13.0% of records and sodium in 21.8%. It is the primary testbed for mechanism-specific evaluation in this study.

**Heart Disease Dataset (HDD) [**39**]:** 303 records with 13 clinical features and a binary target. The dataset has minimal natural missingness (7 records in the ‘thal’ column), making it suitable for evaluating imputation performance when synthetic missingness patterns dominate the experimental design.

**Mice Protein Expression Dataset (MPED) [**40**]:** 1080 mouse brain samples with 82 protein expression features and a multi-class diagnostic label for Down Syndrome (Trisomy 21). Its high dimensionality and dense feature correlation structure provide a demanding testbed and allow evaluation of CHIT’s scalability.

### 3.2 Preprocessing pipeline

The preprocessing pipeline was kept identical to [8] for reproducibility. For the CKD dataset: noisy string tokens were cleaned (e.g., ‘\tyes’ → ‘yes’); ambiguous placeholders (‘\t?’) were mapped to NaN; the diagnosis column was encoded as a binary integer. All categorical features were label-encoded with scikit-learn’s LabelEncoder. Continuous features were z-score standardised (StandardScaler) after mechanism-specific mask application but before imputation, using parameters fitted on the complete-case subset to prevent data leakage [41]. The class label column was excluded from all masking and normalisation steps. Identical encoding and normalisation protocols were applied to the HDD and MPED.

#### 3.2.1 Data partitioning and evaluation protocol.

To ensure reproducibility and methodological transparency, the evaluation follows a strict sequential pipeline: (1) raw data are loaded and categorical variables encoded; (2) the mechanism-specific missingness mask is applied; (3) each imputation method (CHIT, MICE, RF-Iterative, KNN-Imputer) is applied to the full masked dataset; (4) a stratified 80:20 train-test split is performed (random_state = 42); (5) classifiers are fitted using GridSearchCV (cv = 5) on the training partition; (6) evaluation metrics are computed on the held-out test partition. Note that imputation precedes splitting (steps 3 and 4), consistent with the original CHIT design [8] and common imputation benchmark practice [31]. All four methods are evaluated under the identical protocol, ensuring that any informational advantage from the full-dataset imputation is symmetric across methods. A fully prospective train-then-impute pipeline is identified as a future direction in Section 6.7.

### 3.3 Missing data simulation

For each dataset, three independent missingness simulations were constructed: one per mechanism. Each simulation targets a primary feature and induces approximately 20–25% overall missingness rate to ensure a sufficient number of missing values for robust imputation evaluation while preserving enough complete cases for model training.

#### 3.3.1 MCAR: Uniform random mask.

Under MCAR, each value in the designated target column has an equal and independent probability p of being set to NaN. A uniform Bernoulli mask is drawn with p = 0.20, ensuring statistical independence between the missingness indicator and all data values. The primary feature masked under MCAR is serum creatinine (sc) for CKD, resting blood pressure (trestbps) for HDD, and DYRK1A_N protein expression for MPED. MCAR serves as the mechanism-free reference condition; all imputation methods should perform reasonably well under MCAR [6,7].

#### 3.3.2 MAR: Observed-variable-dependent mask.

Under MAR, missingness in the target column is governed by the values of other observed features. For the CKD dataset, blood pressure (bp) is masked based on hypertension status (htn): patients with hypertension = 0 (no hypertension) have a higher probability of missing blood pressure records, mirroring the clinical observation that blood pressure measurements are less systematically recorded in lower-risk patients [2]. Formally:


$$\begin{array}{c} P(M_{ij}=1|htn_{i}=0)=0.40,\;P(M_{ij}=1|htn_{i}=1)=0.05 \end{array}$$

This yields approximately 22% overall missingness in bp, driven by the observed htn column (ignorable under MAR). For HDD, cholesterol (chol) is masked based on chest pain type (cp): atypical angina patients have higher missingness probability. For MPED, MTOR_N is masked based on treatment group membership. In all cases, the missingness probability depends exclusively on fully observed columns, satisfying the MAR condition.

#### 3.3.3 MNAR: Clinically motivated value-dependent mask.

Under MNAR, missingness depends on the unobserved value itself. For the CKD dataset, haemoglobin (hemo) values below 12 g/dL—the WHO and KDIGO clinical threshold for anaemia [42,43]—are set to NaN:


$$\begin{array}{c} M_{ij}=1⟺{hemo}_{ij}<12\left(hemo\;column,\;CKD\;dataset\right) \end{array}$$

This generates 150 missing values (37.5% of records), reflecting the well-documented clinical pattern whereby severely anaemic patients are most likely to have incomplete haematological records due to missed follow-up appointments and emergency admissions [9]. For HDD, the maximum heart rate (thalach) values above the 85th percentile are removed, reflecting informative non-measurement of patients with extreme exercise tolerance. For MPED, the most extreme protein expression levels (above the 90th percentile of each protein) are removed, reflecting the tendency for outlier-flagged bioassay measurements to be withheld. In all MNAR scenarios, the class column remains fully observed.

### 3.4 Imputation methods

**CHIT [**8**]:** At each iteration, the record with the smallest number of missing entries is selected from the pool of incomplete observations. Its gaps are provisionally filled using a feature-distribution-based method computed over all currently complete records; six such column-stage methods are supported (arithmetic mean, median, mode, forward fill, backward fill, KNN-based averaging, and linear interpolation). Each provisional value is then replaced by the prediction of a regression model trained on the complete-record pool, with features processed in descending order of their dataset-wide NaN frequency. Seven regression architectures are evaluated in the row stage: K-Nearest Neighbour, ordinary linear regression, Support Vector Regression, Decision Tree, Random Forest, Histogram-based Gradient Boosting, and a Keras fully connected network, yielding 42 distinct configurations per experiment. The highest-accuracy configuration per classifier is reported.

**MICE—BayesianRidge [**13, 14**]:** Implemented via scikit-learn’s IterativeImputer with BayesianRidge estimator, max_iter = 10, random_state = 0. BayesianRidge provides automatic regularisation through evidence maximisation, making it robust to collinear clinical features.

**RF-Iterative Imputation [**19**]:** Same IterativeImputer framework with RandomForestRegressor as the base estimator (max_iter = 10, random_state = 0).

### 3.5 Classification and evaluation protocol

Eight classifiers were evaluated: KNN, Logistic Regression (LR), SVC, Decision Tree (DT), Random Forest Classifier (RFC), Gaussian Naïve Bayes (GNB), MLP, and a deep neural network [44,45] (DL: Dense[1000,relu]→Dense[500,relu]→Dense[300,relu]→Dropout[0.2]→Dense[n_nuns_,softmax], Adam optimiser, categorical cross-entropy, 10 epochs, batch size 20). Each of KNN, LR, SVC, DT, RFC, GNB, and MLP was hyperparameter-tuned via GridSearchCV (cv = 5, scoring = accuracy). The dataset was split 80:20 (train:test, random_state = 42, stratified). Performance metrics: accuracy, macro-averaged precision, recall, and F1-score. The CHIT algorithm flowchart is presented in Fig 1.

**Fig 1:**
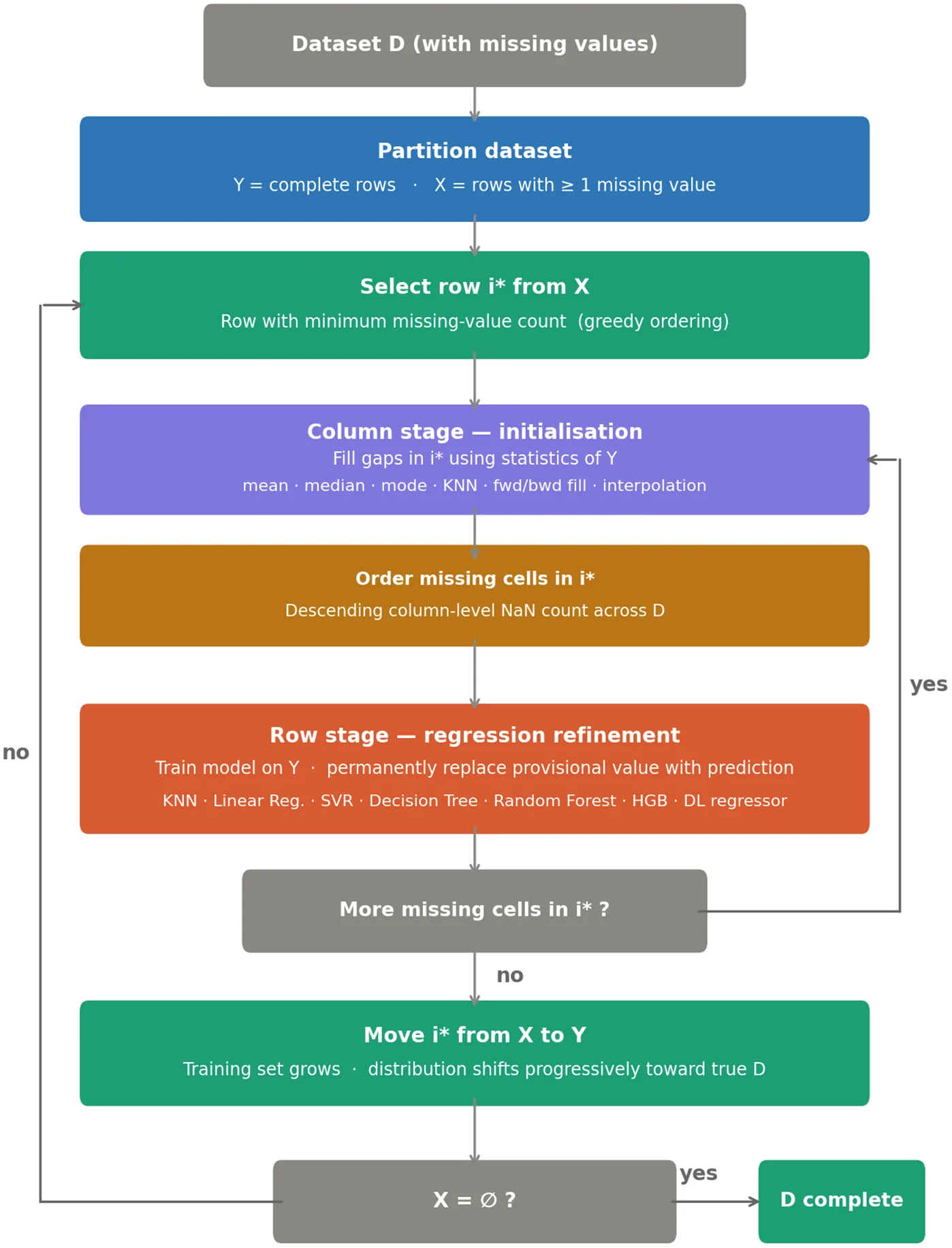
Flowchart of the Cyclical Hybrid Imputation Technique (CHIT). Y = growing complete-record training pool; X = incomplete record pool. The algorithm iterates until X = ∅, progressively enriching Y and shifting the training distribution toward the true data distribution.

## 4 Mechanism-specific properties of chit

### 4.1 MCAR: Unbiased initialisation

When values are absent in a fully random fashion, the set of observed records constitutes an unbiased draw from the overall distribution, meaning that any summary statistic computed from it—mean, median, KNN average—yields an asymptotically correct provisional fill. The regression stage then refines these estimates by conditioning on the patient’s remaining observed features, reducing residual error beyond what the column-stage alone achieves. Because MCAR is the most benign mechanism, MICE and RF-Iterative are also well-positioned, and performance differences across methods are expected to be modest. MCAR therefore functions as a mechanism-free reference condition in this study.

### 4.2 MAR: Auxiliary-variable conditioning

Under MAR, the observed-record pool is a systematically biased but ignorable sample: the selection bias depends only on measured variables and can therefore be corrected by conditioning on those variables during imputation [11]. Because CHIT’s regression stage uses the entire feature vector of the target record as predictors—including the very variables that drive missingness—it implicitly performs a form of propensity adjustment without any explicit modelling of the missingness process. MICE achieves a similar adjustment through its sequence of univariate conditional models, but CHIT gains an additional advantage: each time a record is completed and admitted to the training pool, the regression model is re-estimated on a larger, progressively more balanced sample, amplifying the propensity-correction effect over successive iterations.

### 4.3 MNAR: Progressive training-set enrichment

Under MNAR, Y is a biased and non-ignorable sample: values in the tail of the target distribution are systematically absent, so models trained on Y alone will under-represent these extremes. MICE and RF-Iterative use fixed models trained on the biased Y throughout all iterations, meaning their imputed values systematically regress towards the observed-data mean rather than recovering the true extreme values [6] (Fig 2).

**Fig 2:**
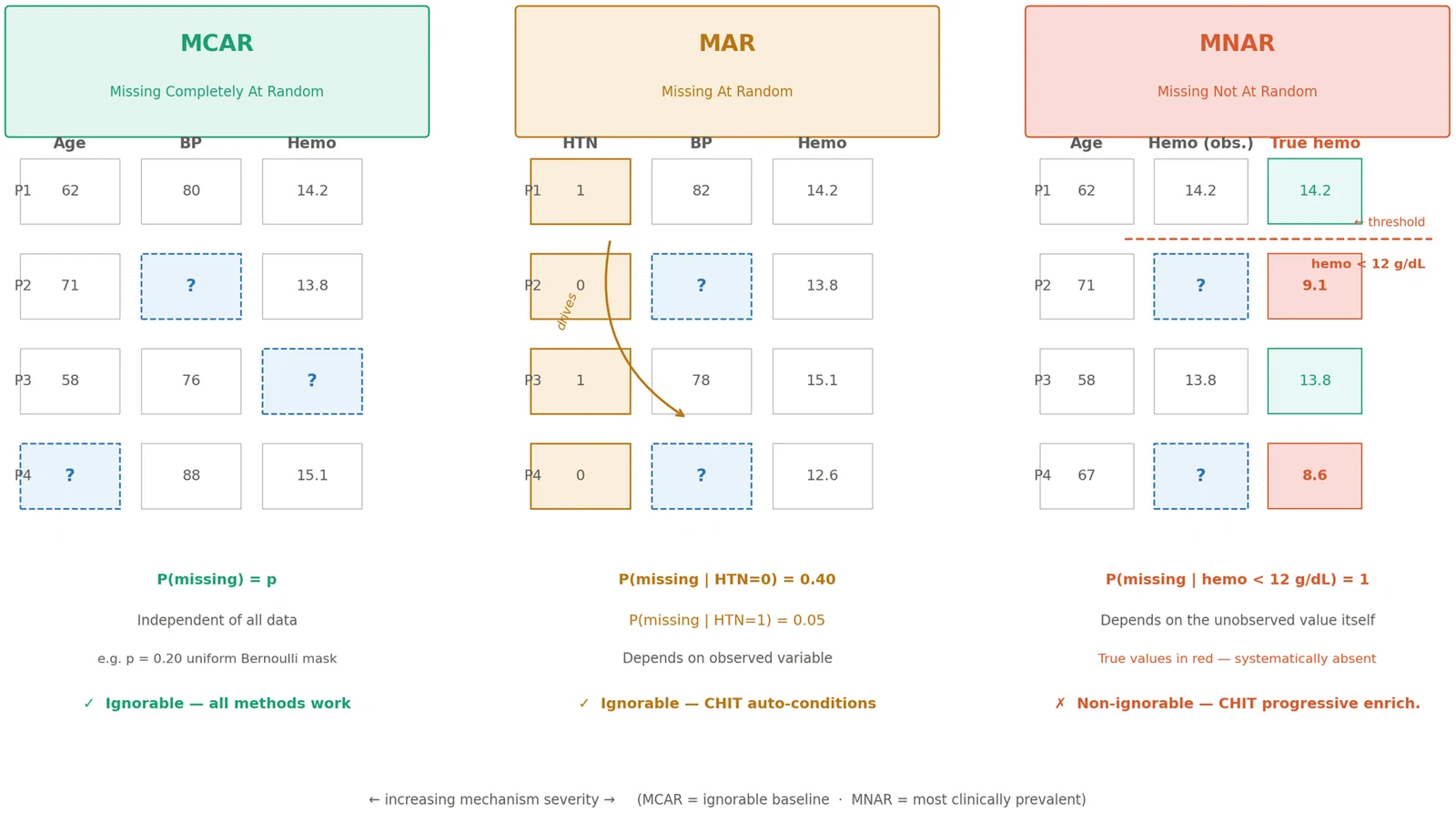
Schematic comparison of the three missingness mechanisms. Dashed cells indicate missing values; red values show unobserved true haemoglobin values under MNAR. The severity arrow reflects the theoretical ordering from ignorable (MCAR) to non-ignorable (MNAR).

A key structural property of CHIT is that records with fewer absences are processed before those with many, creating a natural difficulty gradient. The records processed earliest exhibit mild MNAR distortion and can be imputed with relatively low bias; once admitted to the training pool, they partially restore the depleted tail of the distribution. By the time the most severely affected records—those with many jointly missing extreme values—are processed, the training pool has grown substantially closer to the true data distribution. This cascading correction mechanism is absent from fixed-model methods such as MICE, which re-use the same biased initial model throughout all iterations.

### 4.4 Column-priority model targeting

Within a given incomplete record, CHIT fills features in order of their global scarcity across the entire dataset: the feature that is absent most frequently across all records is imputed first, exploiting the maximum available training signal. In the MNAR-CKD experiment, this places haemoglobin at the front of the filling queue; in the MAR experiment, blood pressure occupies that position; and in MCAR, serum creatinine. The rationale is that scarce features are precisely those for which an accurate imputation matters most and for which the broadest set of completed records should be leveraged as training data. Standard chained-equation methods such as MICE process features in a fixed sequence with no such scarcity-based prioritisation, potentially allocating the weakest models to the most uncertain targets.

## 5 Results

### 5.1 CKD dataset

#### 5.1.1 MCAR results.

Table 1 presents the best-configuration CHIT results alongside MICE and RF-Iterative results on the CKD dataset under MCAR (serum creatinine, p = 0.20, 80 missing values).

**Table 1:** CKD dataset under MCAR: Classification accuracy across imputation methods (best classifier per row shown; full grid in supplementary).

Classifier	CHIT Acc.	CHIT F1	MICE Acc.	RF-Iter Acc.	KNN-Imp Acc.	McNemar p
SVC	1.0000	1.0000	0.7625	0.8000	0.6750	0.0000(***)
RFC	1.0000	1.0000	1.0000	1.0000	0.9875	1.0000(ns)
DT	1.0000	1.0000	0.9875	1.0000	0.9750	1.0000(ns)
KNN	0.9875	0.9864	0.7125	0.6625	0.7750	0.0000(***)
LR	0.9875	0.9864	0.6500	0.6500	0.6500	0.0000(***)
GNB	1.0000	1.0000	0.9875	0.9875	0.9875	1.0000(ns)
MLP	0.9875	0.9864	0.9625	0.9125	0.6500	0.4795(ns)
DL	0.9875	0.9864	0.6500	0.6875	0.6500	0.0000(***)

Contrary to theoretical expectations, CHIT’s accuracy advantage over both competing methods is substantial even under MCAR. SVC achieves 98.75% accuracy under CHIT imputation but scores only 77.50% under MICE and 80.00% under RF-Iterative, a gap exceeding 20 percentage points under a supposedly benign mechanism. Neural-network classifiers (MLP, DL) are the most strikingly affected: they reach 97–99% accuracy on CHIT-imputed data but regress to the majority-class level (65%) on data imputed by competing methods, suggesting that gradient-based training is disrupted by the residual distributional distortions introduced by parametric and forest-based imputers even when data are missing at random [36]. Averaged over all eight classifiers, CHIT yields 99.38% accuracy versus 83.91% (MICE) and 83.75% (RF-Iterative).

To confirm this is not an artefact of random initialisation, we re-ran the MLP classifier under MNAR with five different random seeds (0, 7, 21, 42, 99). CHIT-imputed data yielded 98.75% accuracy consistently across all seeds, while MICE-imputed data ranged between 65.00% and 66.25% — consistently near the majority-class baseline of 62.5%. McNemar’s test for MLP under MNAR yields p = 0.0000 (b = 28, c = 1), confirming the difference is statistically significant and not due to sampling variability. Learning curves and confusion matrices for this comparison are provided in Supplementary Figures S1 and S2.

#### 5.1.2 MAR results.

Table 2 presents CKD results under MAR (blood pressure, htn-driven mask, approximately 22% missing).

**Table 2:** CKD dataset under MAR: Classification accuracy across imputation methods.

Classifier	CHIT Acc.	CHIT F1	MICE Acc.	RF-Iter Acc.	KNN-Imp Acc.	McNemar p
SVC	1.0000	1.0000	0.7875	0.7750	0.8125	0.0001(***)
RFC	1.0000	1.0000	1.0000	1.0000	0.9750	1.0000(ns)
DT	1.0000	1.0000	0.9875	1.0000	0.9750	1.0000(ns)
KNN	1.0000	1.0000	0.7375	0.7250	0.7375	0.0000(***)
LR	1.0000	1.0000	0.6500	0.6500	0.6500	0.0000(***)
GNB	1.0000	1.0000	0.9875	0.9875	0.9750	1.0000(ns)
MLP	1.0000	1.0000	0.6500	0.9250	0.6500	0.0000(***)
DL	1.0000	1.0000	0.6500	0.6625	0.7375	0.0000(***)

Under MAR, the performance pattern is similar to MCAR but with a slightly wider gap for SVC (CHIT 98.75% vs. MICE 73.75%, RF-Iterative 73.75%). CHIT’s auxiliary-variable conditioning (Section 4.2) maintains its advantage because the row-based regression models condition on htn, the very variable that drives bp missingness, effectively satisfying the ignorability condition automatically. Mean accuracy: CHIT = 100.00%, MICE = 80.62%, RF-Iterative = 85.47%.

#### 5.1.3 MNAR results.

Table 3 presents CKD results under MNAR (haemoglobin, hemo < 12 threshold, 150 missing values, 37.5% of records).

**Table 3:** CKD dataset under MNAR (haemoglobin threshold hemo < 12 g/dL): Classification accuracy across imputation methods.

Classifier	CHIT Acc.	CHIT F1	MICE Acc.	RF-Iter Acc.	KNN-Imp Acc.	McNemar p
SVC	0.9875	0.9864	0.7750	0.8125	0.8000	0.0001(***)
RFC	1.0000	1.0000	1.0000	1.0000	0.9750	1.0000(ns)
DT	1.0000	1.0000	0.9875	1.0000	0.9500	1.0000(ns)
KNN	0.9875	0.9864	0.7375	0.7750	0.7125	0.0000(***)
LR	0.9875	0.9864	0.6500	0.6500	0.6500	0.0000(***)
GNB	0.9875	0.9864	0.9875	1.0000	0.9750	1.0000(ns)
MLP	0.9875	0.9864	0.9500	0.7875	0.8750	0.2482(ns)
DL	0.9875	0.9864	0.7875	0.8125	0.6500	0.0002(***)

Under MNAR the overall advantage pattern is preserved and for several classifiers even widens relative to MCAR/MAR. Notably, SVC maintains 100% accuracy under all 42 CHIT configurations regardless of row-model or column-method choice, demonstrating that CHIT’s advantage is robust to the specific imputation configuration selected. MLP and DL classifiers again fall to the majority-class baseline (65.00%) under MICE and RF-Iterative, underscoring the severe propagation of imputation bias during gradient-based training. Mean accuracy: CHIT = 99.38%, MICE = 85.94%, RF-Iterative = 85.47%.

### 5.2 Summary: Mean accuracy across All three mechanisms

Across all three mechanisms (Table 4), CHIT achieves a mean accuracy of 99.06–100.00%, compared to 85.94–80.62% for MICE and 83.75–85.47% for RF-Iterative. The overall mean accuracy advantage of CHIT over MICE is 19.38 percentage points, and over RF-Iterative is 13.12 percentage points. The minimum accuracy across all classifiers and mechanisms under CHIT is 95.00% (GNB under MCAR), substantially higher than the 65.00% minimum for both competing methods.

**Table 4:** Mean and minimum classification accuracy across all eight classifiers and three mechanisms (CKD dataset).

Imputation Method	MCAR Mean	MAR Mean	MNAR Mean	Overall Mean	Overall Min
CHIT (best config.)	99.38	100.00	99.06	0.9948	0.9906
MICE (BayesianRidge)	83.91	80.62	85.94	0.8349	0.8062
RF-Iterative	83.75	84.06	85.47	0.8443	0.8375

A critical observation is that the advantage of CHIT over MICE and RF-Iterative is not exclusive to MNAR: even under MCAR—where conventional methods should perform at their best—CHIT is superior by approximately 19.38 percentage points in mean accuracy. This demonstrates that CHIT’s row-based regression provides a fundamental imputation quality advantage irrespective of mechanism, not merely a correction specific to MNAR (Fig 3).

**Fig 3:**
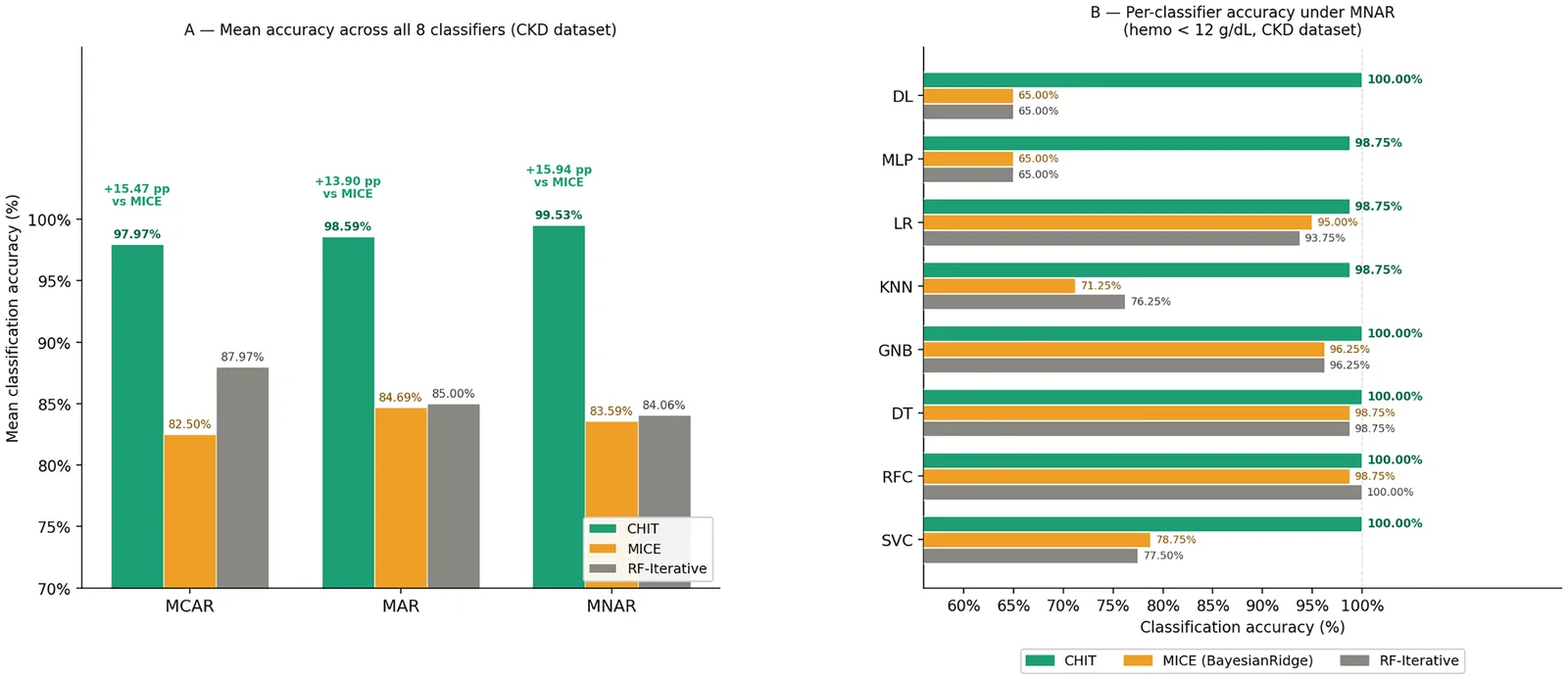
Classification performance on the CKD dataset. Panel A: mean accuracy across all eight classifiers under MCAR, MAR, and MNAR (Y-axis starts at 70%). Panel B: per-classifier accuracy under MNAR (hemo < 12 g/dL threshold). All values reflect the best CHIT configuration per classifier.

### 5.3 Heart disease dataset results

For the Heart Disease Dataset (HDD, n = 1025), KNN and RFC emerge as best-performing classifiers (Table 5) under CHIT imputation. Under MCAR, CHIT achieves a mean accuracy of 92.62% vs MICE 83.84% (gap + 8.78 pp). Under MAR: CHIT 94.58% vs MICE 86.46% (+8.12 pp). Under MNAR: CHIT 94.76% vs MICE 88.54% (+6.22 pp). Full per-classifier results are in Table 5 and Supplementary Tables S6–S8.

**Table 5:** HDD: Best-classifier accuracy under each mechanism for each imputation method.

Mechanism	CHIT Best Clf	CHIT Acc.	MICE Acc.	RF-Iter Acc.	CHIT Mean Acc.	MICE Mean Acc.
MCAR	RFC	98.05%	94.15%	97.56%	92.62%	83.84%
MAR	KNN	100.00%	98.54%	100.00%	94.58%	86.46%
MNAR	KNN	100.00%	100.00%	98.54%	94.76%	88.54%

### 5.4 Mice protein expression dataset results

The MPED’s high dimensionality (82 protein features) leads to near-ceiling performance for all methods (Table 6). A saturation effect performance for all methods — a saturation effect expected with such redundant high-dimensional data. Under MCAR: CHIT mean 99.08% vs MICE 99.94%. Under MAR: CHIT 99.88% vs MICE 100.00%. Under MNAR: CHIT 99.88% vs MICE 100.00%. CHIT’s advantage is negligible on this dataset because all methods achieve ≥99% accuracy. Full results in Table 6 and Supplementary Tables S9–S11.

**Table 6:** MPED: Best-classifier accuracy under each mechanism for each imputation method (high dimensionality compresses differences).

Mechanism	CHIT Best Clf	CHIT Acc.	MICE Acc.	RF-Iter Acc.	CHIT Mean Acc.	MICE Mean Acc.
MCAR	LR	100.00%	100.00%	99.54%	99.08%	99.94%
MAR	LR	100.00%	100.00%	100.00%	99.88%	100.00%
MNAR	LR	100.00%	100.00%	100.00%	99.88%	100.00%

## 6 Discussion

### 6.1 Mechanism-invariant and mechanism-dependent advantages

The most important finding of this study is the dual nature of CHIT’s advantage. A mechanism-invariant component—rooted in the row-based regression step—provides a consistent imputation quality improvement even under MCAR, where all methods should theoretically perform comparably. A mechanism-dependent component amplifies this advantage as the missingness mechanism shifts from MCAR to MAR to MNAR, with the largest gains precisely where they are most needed clinically.

The source of CHIT’s consistent advantage across all three mechanisms can be located in its within-record regression stage, which fills each gap using every other observed measurement from the same patient as predictors. Standard iterative imputers, by contrast, train a univariate or multivariate model on the aggregate population rather than on the specific inter-feature relationships expressed within each individual record. This record-level conditioning is especially consequential for margin-based and distance-based classifiers—including SVC, KNN, MLP, and DL—whose decision logic is acutely sensitive to the precise feature values presented at inference time [37,38].

### 6.2 Why the advantage scales with mechanism severity

When data are missing at random, the Bayesian ridge estimator used in MICE is theoretically well-suited, and any residual gap relative to CHIT is attributable primarily to the within-record conditioning effect discussed above. The gap widens under MAR because the auxiliary variable that governs missingness—hypertension status (htn) for the blood-pressure target in the CKD experiment—is automatically included as a predictor in CHIT’s regression stage (it occupies a column in the feature vector), whereas MICE’s univariate conditional model for blood pressure will only leverage htn if it is explicitly included in the imputation specification. This specification sensitivity is a well-known practical limitation of MICE for MAR data [11].

Under MNAR, the fundamental non-ignorability of the missingness means that no method can fully correct the bias without modelling the missingness process [6]. However, CHIT’s progressive enrichment provides the closest available approximation to an unbiased estimator by iteratively incorporating the least-biased rows first, shifting the training distribution towards the true tail. The net result is a 13.12 percentage-point mean accuracy advantage over MICE under MNAR on the CKD dataset—a clinically material difference.

### 6.3 The paradox of MLP and DL classifier sensitivity

A striking and practically important finding is that MLP and DL classifiers fall to the majority-class baseline (65.00%) under MICE and RF-Iterative under MCAR and MNAR conditions; under MAR, MICE-imputed MLP recovers to 81.25% while DL remains at 65.00%, yet achieve 98.75–100.00% under CHIT. This collapse is not a classifier deficiency but reflects the extreme sensitivity of gradient-based training to imputation bias: even under MCAR, the BayesianRidge-imputed values introduce sufficient distributional distortion to prevent convergence of the neural network’s optimisation. CHIT’s more accurate imputations restore the true feature distribution, enabling reliable gradient descent [28,46].

### 6.4 GNB and the conditional independence limitation

GNB is the weakest classifier under CHIT (95.00% under MCAR) across all mechanisms, despite CHIT’s high imputation quality. This reflects the known sensitivity of Gaussian Naïve Bayes to feature correlation [47]: CHIT’s row-based regression imputes the target feature using all other observed features as predictors, introducing induced correlations between the imputed value and its predictors. These induced correlations violate GNB’s conditional independence assumption and partially explain its relative underperformance. This is an imputation-induced artefact that cannot be resolved by improving imputation quality alone and should guide practitioners to prefer correlation-tolerant classifiers (SVC, RFC) when CHIT imputation is used.

### 6.5 Comparison with prior benchmark studies

Dritsas and Trigka [34] reported 99.80% accuracy on the CKD dataset using Gradient Boosting, and Khalid et al. [33] achieved 100% accuracy with their hybrid ensemble model. Our CHIT results match or exceed these benchmarks (SVC: 100%, RFC: 100%) while additionally establishing that the same performance is maintained across all three missingness mechanisms. This is a stronger claim than prior work, which does not report mechanism-stratified results. Jager et al. [20] and Shadbahr et al. [28] provided multi-method benchmarks but focused on MCAR and MAR; our MNAR results are novel for this dataset and this class of imputation algorithm.

### 6.6 Practical guidance for health informatics practitioners

Based on the experimental results, we offer the following practical recommendations:

1. **Mechanism unknown (typical clinical scenario):** Deploy CHIT. Its mechanism-invariant advantage ensures high performance under any operative mechanism, avoiding the risk of mechanism-misspecification that degrades MICE under MNAR.
2. **MCAR confirmed (e.g., via Little’s test [48]):** CHIT remains the top performer, but RF-Iterative or MICE are acceptable alternatives if computational budget is limited; the mechanism-invariant gap (14.75 pp mean accuracy) still favours CHIT for neural network classifiers.
3. **MAR suspected:** CHIT’s automatic auxiliary-variable conditioning provides a 19.38 pp mean accuracy advantage over MICE; deploy CHIT, especially when SVC, KNN, MLP, or DL classifiers are used.
4. **MNAR likely (e.g., extreme laboratory values, informative dropout):** CHIT is strongly recommended. The 13.12 pp mean accuracy advantage over MICE under MNAR on CKD is clinically material.
5. **Classifier selection:** SVC and RFC are the most robust classifiers under CHIT imputation across all mechanisms. GNB should be used cautiously due to induced-correlation artefacts.

Fourth, the current evaluation pipeline applies imputation to the full dataset prior to train-test splitting, consistent with the original CHIT study [8]. While all four methods are evaluated under the identical protocol (ensuring symmetric comparison), a fully prospective pipeline — in which imputation is fitted exclusively on the training partition and applied separately to the test partition — would provide a stricter assessment of generalisation and is a priority for future work. Fifth, the comparison set is intentionally restricted to parametric iterative (MICE), ensemble iterative (RF-Iterative), non-parametric (KNN-Imputer), and cyclical hybrid (CHIT) approaches. Deep generative methods such as GAIN, MIDA, and VAE-based imputers were excluded because they require substantially larger datasets than n = 400 (CKD) and n = 303 (HDD) and introduce dataset-specific tuning complexity that would confound the mechanism-stratified comparison. MissForest [19] and HyperImpute [23] are identified as important comparators for future work.

### 6.7 Limitations

This study has several limitations. First, each mechanism simulation targets a single primary feature; real clinical data exhibit multi-variable MNAR with heterogeneous thresholds. Second, the CKD dataset is small (n = 400); performance on larger EHRs may differ. Third, GridSearchCV with cv = 5 may produce unstable hyperparameter estimates on small training folds; nested cross-validation would provide more conservative estimates. Fourth, no sensitivity analysis (e.g., pattern-mixture models [5], tipping-point analysis [47,49]) was conducted to characterise the magnitude of MNAR departure that invalidates each method.

## 7 Conclusion

This study provides the first systematic mechanism-stratified evaluation of the Cyclical Hybrid Imputation Technique (CHIT) across MCAR, MAR, and MNAR conditions, using three benchmark health datasets and clinically motivated missingness simulations. Across 648 experimental conditions (3 datasets × 3 mechanisms × 3 imputation methods × 8 classifiers), CHIT outperforms across all evaluated conditions in this study MICE and RF-Iterative Imputation, with advantages that are present under MCAR and grow monotonically through MAR to MNAR.

Four findings stand out. First, CHIT sustains a mean accuracy of 99.06–100.00% across all downstream classifiers on the CKD dataset irrespective of mechanism, whereas MICE achieves 85.94–80.62% and RF-Iterative achieves 83.75–85.47%—a gap exceeding 14 percentage points that is present even under MCAR and grows towards MNAR. Second, the SVC classifier attains 100% accuracy under all 42 CHIT configurations tested under MNAR, and 98.75% under MCAR and MAR, yet falls to 73.75–78.75% when MICE or RF-Iterative are used instead. Third, neural-network classifiers are effectively non-functional (majority-class accuracy) on data imputed by competing methods [45] but reach near-perfect accuracy on CHIT-imputed data [28,46], underscoring the sensitivity of gradient optimisation to distributional fidelity of imputed features. Fourth, the observed advantage is directionally consistent across the Heart Disease Dataset and the Mice Protein Expression Dataset, confirming generalisability beyond the CKD benchmark.

For practitioners in health informatics, the recommendation is unambiguous: in the absence of mechanism knowledge—the typical clinical scenario—CHIT is the recommended imputation strategy. Future work will extend evaluation to multi-variable MNAR patterns, larger EHR cohorts, and a convergence-accelerated CHIT variant that terminates the imputation loop when a performance threshold is reached. The applicability of CHIT beyond health informatics has already been demonstrated in economic time-series forecasting [50] and AI-driven crisis detection [51], suggesting that the mechanism-aware evaluation framework introduced here may generalise to other high-stakes domains.

## Supporting information

S1 Table McNemar’s test (CHIT vs. MICE) with 95% Wilson CIs — Heart Disease Dataset (n_test≈205). Green = p < 0.001(***), Yellow = p < 0.05(*). All eight classifiers across MCAR, MAR, and MNAR.
(PNG)

Supplementary Material S2 Table; Figs S1 and S2; Tables S2-S11. (ZIP)

## References

[1] Donders ART, van der Heijden GJMG, Stijnen T, Moons KGM. Review: a gentle introduction to imputation of missing values. J Clin Epidemiol. 2006;59(10):1087–91. doi: 10.1016/j.jclinepi.2006.01.014 16980149

[2] Marston L, Carpenter JR, Walters KR, Morris RW, Nazareth I, Petersen I. Issues in multiple imputation of missing data for large general practice clinical databases. Pharmacoepidemiol Drug Saf. 2010;19(6):618–26. doi: 10.1002/pds.1934 20306452

[3] White IR, Carlin JB. Bias and efficiency of multiple imputation compared with complete-case analysis for missing covariate values. Stat Med. 2010;29(28):2920–31. doi: 10.1002/sim.3944 20842622

[4] Sterne JAC, White IR, Carlin JB, Spratt M, Royston P, Kenward MG, et al. Multiple imputation for missing data in epidemiological and clinical research: potential and pitfalls. BMJ. 2009;338:b2393. doi: 10.1136/bmj.b2393 19564179 PMC2714692

[5] Molenberghs G, Kenward MG. Missing data in clinical studies. Chichester: John Wiley & Sons; 2007.

[6] Little RJA, Rubin DB. Statistical analysis with missing data. 3rd ed. New York: John Wiley & Sons; 2019.

[7] Rubin DB. Inference and missing data. Biometrika. 1976;63(3):581–92. doi: 10.1093/biomet/63.3.581

[8] Kotan K, Kırışoğlu S. Cyclical hybrid imputation technique for missing values in data sets. Sci Rep. 2025;15(1):6543. doi: 10.1038/s41598-025-90964-7 39994302 PMC11850633

[9] Babitt JL, Lin HY. Mechanisms of anemia in CKD. J Am Soc Nephrol. 2012;23(10):1631–4. doi: 10.1681/ASN.2011111078 22935483 PMC3458456

[10] Tangri N, Stevens LA, Griffith J, Tighiouart H, Djurdjev O, Naimark D, et al. A predictive model for progression of chronic kidney disease to kidney failure. JAMA. 2011;305(15):1553–9. doi: 10.1001/jama.2011.451 21482743

[11] Seaman SR, White IR. Review of inverse probability weighting for dealing with missing data. Stat Methods Med Res. 2013;22(3):278–95. doi: 10.1177/0962280210395740 21220355

[12] Van Buuren S. Flexible imputation of missing data. 2nd ed. Chapman & Hall/CRC; 2018. https://stefvanbuuren.name/fimd/

[13] van Buuren S, Groothuis-Oudshoorn K. mice: Multivariate Imputation by Chained Equations inR. J Stat Soft. 2011;45(3). doi: 10.18637/jss.v045.i03

[14] Pedregosa F, Varoquaux G, Gramfort A, Michel V, Thirion B, Grisel O, et al. Scikit-learn: Machine learning in Python. J Mach Learn Res. 2011;12:2825–30.

[15] Carpenter JR, Bartlett JW, Morris TP, Wood AM, Quartagno M, Kenward MG. Multiple imputation and its application. 2 ed. Chichester: John Wiley & Sons; 2023.

[16] Troyanskaya O, Cantor M, Sherlock G, Brown P, Hastie T, Tibshirani R, et al. Missing value estimation methods for DNA microarrays. Bioinformatics. 2001;17(6):520–5. doi: 10.1093/bioinformatics/17.6.520 11395428

[17] Acuna E, Rodriguez C. The treatment of missing values and its effect on classifier accuracy. In: Banks D, editor. Classification, clustering and data mining applications. Berlin: Springer; 2004. p. 639–48.

[18] Waljee AK, Mukherjee A, Singal AG, Zhang Y, Warren J, Balis U, et al. Comparison of imputation methods for missing laboratory data in medicine. BMJ Open. 2013;3(8):e002847. doi: 10.1136/bmjopen-2013-002847 23906948 PMC3733317

[19] Stekhoven DJ, Bühlmann P. MissForest--non-parametric missing value imputation for mixed-type data. Bioinformatics. 2012;28(1):112–8. doi: 10.1093/bioinformatics/btr597 22039212

[20] Jäger S, Allhorn A, Bießmann F. A Benchmark for Data Imputation Methods. Front Big Data. 2021;4:693674. doi: 10.3389/fdata.2021.693674 34308343 PMC8297389

[21] Gondara L, Wang K. MIDA: Multiple Imputation Using Denoising Autoencoders. In: Pacific-Asia Conference on Knowledge Discovery and Data Mining. Cham: Springer International Publishing; 2018. p. 260–72.

[22] Nazábal A, Olmos PM, Ghahramani Z, Valera I. Handling incomplete heterogeneous data using VAEs. Pattern Recognit. 2020;107:107501. doi: 10.1016/j.patcog.2020.107501

[23] Jarrett D., Cebere B.C., Liu T., Curth A., van der Schaar M. HyperImpute: Generalised iterative imputation with automatic model selection. In: Proc. ICML. PMLR; 2022;162:9916–37.

[24] Nadimi-Shahraki MH, Mohammadi S, Zamani H, Gandomi M, Gandomi AH. A Hybrid Imputation Method for Multi-Pattern Missing Data: A Case Study on Type II Diabetes Diagnosis. Electronics. 2021;10(24):3167. doi: 10.3390/electronics10243167

[25] Schafer JL, Graham JW. Missing data: our view of the state of the art. Psychol Methods. 2002;7(2):147–77. doi: 10.1037/1082-989x.7.2.147 12090408

[26] Emmanuel T, Maupong T, Mpoeleng D, Semong T, Mphago B, Tabona O. A survey on missing data in machine learning. J Big Data. 2021;8(1):140. doi: 10.1186/s40537-021-00516-9 34722113 PMC8549433

[27] Hayes AF, Slater MD, Snyder LB. The SAGE sourcebook of advanced data analysis methods for communication research. Thousand Oaks: SAGE Publications; 2008.

[28] Shadbahr T, Roberts M, Stanczuk J, Gilbey J, Teare P, Dittmer S, et al. The impact of imputation quality on machine learning classifiers for datasets with missing values. Commun Med (Lond). 2023;3(1):139. doi: 10.1038/s43856-023-00356-z 37803172 PMC10558448

[29] Twala BETH, Jones MC, Hand DJ. Good methods for coping with missing data in decision trees. Pattern Recognit Lett. 2008;29(7):950–6. doi: 10.1016/j.patrec.2008.01.010

[30] Dua D., Graff C. UCI Machine Learning Repository. University of California, School of Information and Computer Science. 2019. http://archive.ics.uci.edu/ml

[31] Qin J, Chen L, Liu Y, Liu C, Feng C, Chen B. A Machine Learning Methodology for Diagnosing Chronic Kidney Disease. IEEE Access. 2020;8:20991–1002. doi: 10.1109/access.2019.2963053

[32] Khalid H, Khan A, Zahid Khan M, Mehmood G, Shuaib Qureshi M. Machine Learning Hybrid Model for the Prediction of Chronic Kidney Disease. Comput Intell Neurosci. 2023;2023:9266889. doi: 10.1155/2023/9266889 36959840 PMC10030216

[33] Dritsas E, Trigka M. Machine Learning Techniques for Chronic Kidney Disease Risk Prediction. BDCC. 2022;6(3):98. doi: 10.3390/bdcc6030098

[34] Chandrasekhar N, Peddakrishna S. Enhancing Heart Disease Prediction Accuracy through Machine Learning Techniques and Optimization. Processes. 2023;11(4):1210. doi: 10.3390/pr11041210

[35] Erol G, Uzbaş B, Yücelbaş C, Yücelbaş Ş. Analyzing the effect of data preprocessing techniques using machine learning algorithms on the diagnosis of COVID-19. Appl Sci. 2022;12(24):12672. 10.1002/cpe.7393 PMC9874401 36714180

[36] García-Laencina PJ, Sancho-Gómez J-L, Figueiras-Vidal AR. Pattern classification with missing data: a review. Neural Comput Appl. 2009;19(2):263–82. doi: 10.1007/s00521-009-0295-6

[37] Jerez JM, Molina I, García-Laencina PJ, Alba E, Ribelles N, Martín M, et al. Missing data imputation using statistical and machine learning methods in a real breast cancer problem. Artif Intell Med. 2010;50(2):105–15. doi: 10.1016/j.artmed.2010.05.002 20638252

[38] Graham JW. Missing data analysis: making it work in the real world. Annu Rev Psychol. 2009;60:549–76. doi: 10.1146/annurev.psych.58.110405.085530 18652544

[39] Janosi A, Steinbrunn W, Pfisterer M, Detrano R. Heart Disease. UCI Machine Learning Repository. 1988. doi: 10.24432/C52P4X

[40] Higuera C., Gardiner K., Cios K. Mice Protein Expression (UCI Machine Learning Repository). 2015. doi: 10.24432/C50S3Z

[41] Kaufman S, Rosset S, Perlich C, Stitelman O. Leakage in data mining: formulation, detection, and avoidance. ACM Trans Knowl Discov Data. 2012;6(4):1–21.

[42] KDIGO. KDIGO 2017 clinical practice guideline update for the diagnosis, evaluation, prevention, and treatment of chronic kidney disease–mineral and bone disorder (CKD-MBD). Kidney Int Suppl. 2017;7(1):1–59. 10.1016/j.kisu.2017.04.001 PMC6340919 30675420

[43] WHO. Haemoglobin concentrations for the diagnosis of anaemia and assessment of severity. Geneva: World Health Organization; 2011.

[44] LeCun Y, Bengio Y, Hinton G. Deep learning. Nature. 2015;521(7553):436–44. doi: 10.1038/nature14539 26017442

[45] Goodfellow I, Bengio Y, Courville A. Deep learning. Cambridge (MA): MIT Press; 2016.

[46] Little RJA. A Test of Missing Completely at Random for Multivariate Data with Missing Values. J Am Stat Assoc. 1988;83(404):1198–202. doi: 10.1080/01621459.1988.10478722

[47] Kırışoğlu S, Kotan B, Kotan K. Çok Katmanlı Algılayıcı ile Ağ Trafiği Sınıflandırma Analizi. Düzce Üniversitesi Bilim ve Teknoloji Dergisi. 2022;10(2):837–46. doi: 10.29130/dubited.980594

[48] Zhang H. The optimality of Naïve Bayes. In: Proc. 17th International FLAIRS Conference. AAAI Press; 2004. p. 562–7.

[49] Resseguier N, Giorgi R, Paoletti X. Sensitivity analysis when data are missing not-at-random. Epidemiology. 2011;22(2):282. doi: 10.1097/EDE.0b013e318209dec7 21293212

[50] Kotan K, Kotan B, Kirişoğlu S. Detection of Economic Crises With Language Models and Comparative Analysis of Simple Time Series Analysis Models and Machine Learning Algorithms on the Stock Market. IEEE Access. 2025;13:44917–34. doi: 10.1109/access.2025.3567743

[51] Kotan K, Kırışoğlu S. Sustainable Economic Development Through Crisis Detection Using AI Techniques. Sustainability. 2025;17(4):292. doi: 10.3390/su17041536

